# Multimonth Dispensing of Antiretroviral Therapy Protects the Most Vulnerable From 2 Pandemics at Once

**DOI:** 10.9745/GHSP-D-20-00160

**Published:** 2020-06-30

**Authors:** Ariana Moriah Traub, Temitayo Ifafore-Calfee, Benjamin Ryan Phelps

**Affiliations:** a Sustaining Technical and Analytic Resources, United States Agency for International Development (USAID), Washington, DC, USA.; b Office of HIV/AIDS, USAID, Washington, DC, USA.

## Abstract

We encourage governments in countries that have a high prevalence of people living with HIV to implement multimonth dispensing of antiretroviral therapy to safeguard both patients with HIV and health care workers from coronavirus disease COVID-19.

## Key Messages

- Multimonth dispensing, which provides patients with several months of antiretroviral therapy, benefits HIV patients by decreasing the number of required clinic visits, improving retention, and improving viral suppression.
- With the increased spread of severe acute respiratory syndrome coronavirus 2, we encourage governments, ministries of health, and health care facilities to implement multimonth dispensing for patients with HIV who are stable to reduce clinic visits and safeguard both health care workers and patients from coronavirus disease 2019.

Since the emergence of the severe acute respiratory syndrome coronavirus 2 (SARS-CoV-2) in December 2019, the virus has been identified in more than 183 countries.^1^ Its rapid spread is of major concern for those living in low- and middle-income countries and specifically people living with HIV (PLHIV). With the dearth of studies on HIV-coronavirus disease 2019 (COVID-19) coinfections, countries have no clear evidence on specific risks for PLHIV.^2^^,^^3^ However, as with other infectious diseases, it is expected that PLHIV who are not virally suppressed and/or on antiretroviral therapy (ART) might be at an increased risk of COVID-19 infection, severe disease, and poor health outcomes.^2^^,^^3^ Thus, every precaution must be taken to avoid HIV-COVID-19 coinfection and keep the number of COVID-19 cases at a minimum in countries with high proportions of PLHIV.

To minimize the spread of COVID-19, PLHIV are encouraged to follow public health guidelines to limit in-person interactions and general mitigation recommendations, including social distancing and good hand hygiene. Refilling lifesaving antiretroviral drugs requires interpersonal interaction and brings the potential of increasing the spread of SARS-CoV-2 and other transmissible diseases. Thus, policy makers—including the U.S. President’s Emergency Plan for AIDS Relief—are encouraging countries to provide PLHIV with a multimonth supply of ART as a key strategy to safeguard PLHIV and health care workers involved in providing HIV services.^4^

## MULTIMONTH ART DISPENSING MITIGATES RISK OF HIV AND COVID-19

Multimonth dispensing (MMD) is an aspect of differentiated service delivery that provides patients with either 3 or 6 months of medication and eliminates the need for monthly clinic and/or community facility visits. From the patient’s perspective, MMD has already been shown to reduce the cost of travel, reduce patient burden, and limit the hours of work or school lost.^5^^,^^6^ Importantly, MMD improves adherence and viral suppression.^5^^,^^7^ In the context of the COVID-19 pandemic, this viral suppression strengthens the immune system and likely mitigates the risk of severe COVID-19.^8^ MMD, essential to caring for PLHIV, is arguably even more critical during the COVID-19 pandemic.

Currently, only 21 countries have formal 6-month dispensing policies. Two countries allow clinicians to provide 6-month supply when they deem appropriate, and 8 countries have a 3-month dispensing policy in place. South Africa, the country with the largest number of PLHIV, only has a 2-month dispensing policy in place.

To mitigate the damage caused by SARS-CoV-2 while protecting the health of PLHIV, we encourage countries to rapidly implement 3-month if not 6-month dispensing. As a key strategy to safeguard patients and health care workers providing HIV services, MMD also reduces clinic visits, improves viral suppression, encourages social distancing, and potentially saves patients from the dual threat of SARS-CoV-2 and HIV. As SARS-CoV-2 spreads, patient caseloads are likely to increase across low- and middle-income countries where HIV burden is high and millions of people are stable on effective treatment. Implementing MMD quickly will protect the time and health of existing health care professionals and ready our health systems for imminent threats as well as any that may follow.

## Notes

**Peer Reviewed**
